# Assessment of Antimicrobial and Cytotoxic Activities of Liposomes Loaded with Curcumin and *Lippia origanoides* Essential Oil

**DOI:** 10.3390/biom14070851

**Published:** 2024-07-15

**Authors:** Juan Pablo Bedoya-Agudelo, Jhon Esteban López-Carvajal, Edwin Stiven Quiguanás-Guarín, Nestor Cardona, Leonardo Padilla-Sanabria, Jhon Carlos Castaño-Osorio

**Affiliations:** 1 Molecular Immunology Group (GYMOL), Center of Biomedical Research, Faculty of Health Sciences, Quindío University, Armenia 630003, Colombia; jpbedoya@uniquindio.edu.co (J.P.B.-A.); jelopezc_4@uqvirtual.edu.co (J.E.L.-C.); esquiguanasg@uqvirtual.edu.co (E.S.Q.-G.); jhoncarlos@uniquindio.edu.co (J.C.C.-O.); 2 Group of Investigation in Oral Health, Faculty of Dentistry, Antonio Nariño University, Armenia 630001, Colombia; nestorcardonape@uan.edu.co

**Keywords:** liposome, curcumin, *Lippia origanoides* essential oil, antimicrobial, cytotoxicity

## Abstract

(1) Introduction: Curcumin and *Lippia origanoides* essential oils have a broad spectrum of biological activities; however, their physicochemical instability, low solubility, and high volatility limit their therapeutic use. Encapsulation in liposomes has been reported as a feasible approach to increase the physicochemical stability of active substances, protect them from interactions with the environment, modulate their release, reduce their volatility, improve their bioactivity, and reduce their toxicity. To date, there are no reports on the co-encapsulation of curcumin and *Lippia origanoides* essential oils in liposomes. Therefore, the objective of this work is to prepare and physiochemical characterize liposomes loaded with the mixture of these compounds and to evaluate different in vitro biological activities. (2) Methods: Liposomes were produced using the thin-layer method and physiochemical characteristics were calculated. The antimicrobial and cytotoxic activities of both encapsulated and non-encapsulated compounds were evaluated. (3) Results: Empty and loaded nanometric-sized liposomes were obtained that are monodisperse and have a negative zeta potential. They inhibited the growth of *Staphylococcus aureus* and did not exhibit cytotoxic activity against mammalian cells. (4) Conclusions: Encapsulation in liposomes was demonstrated to be a promising strategy for natural compounds possessing antimicrobial activity.

## 1. Introduction

The skin constitutes the main structural barrier of the body in defense against external agents and has important homeostatic functions. It is also the largest organ, with an extensive surface area of approximately 1.8 m^2^ that is in constant contact and exchange with the external environment, and it is formed by three layers: the epidermis, dermis, and subcutaneous tissue [1,2]. However, different pathogens, including bacteria, fungi, viruses, and parasites, can cause skin diseases. The most common bacterial skin pathogens are *Staphylococcus aureus* and group A β-hemolytic streptococci [3]. There is a constant equilibrium between microorganisms and their host; disturbing this equilibrium results in conditions that favor the development of infection [1]. Skin and soft tissue infections (SSTIs) constitute one of the main reasons for consultations globally. They can affect any layer of the skin, fascia, or muscle, with *S. aureus* being the main etiological agent [4]. Despite being part of our cutaneous microbiota, *S. aureus* is also a common cause of infections both in the community and in healthcare settings. Patients have a 64% greater chance of dying when infection is caused by methicillin-resistant *S. aureus* (MRSA) vs. drug-sensitive *S. aureus* [5].

Unfortunately, the growing antimicrobial resistance compromises the effective treatment of different infections caused by various pathogens, a problem that has been associated with increased morbidity and mortality, as well as higher healthcare costs [4,5,6]. However, the clinical development pipeline for new antimicrobials has been depleted. In 2019, the World Health Organization (WHO) found that only 6 of the 32 antibiotics in clinical development for use against the list of priority pathogens could be classified as innovative [5]. Nevertheless, different global initiatives have been implemented, bringing together various sectors of society and different countries to combat antibiotic resistance and search for new antimicrobial molecules. In searching for molecules with potential antimicrobial activity as a response to this global problem, we have become interested in natural compounds as a possible alternative for the treatment of infections caused by multidrug-resistant microorganisms. Natural compounds are a continuous source of new drugs. Approximately 49% of all small molecules approved by the United States Food and Drug Administration (FDA) between 1940 and 2014 were natural products or their direct derivatives [7]. In 2018, our research group developed a topical ointment comprising a mixture of curcumin (CUR), *Lippia origanoides* essential oil (LEO), and a host defense peptide from a coprophagous beetle, that showed antimicrobial activity in a skin infection model in BALB/c mice. This ointment is protected by an invention patent (NC2018/0009648—Colombia). However, during the development of this ointment, the mixture of curcumin and essential oil was found to enhance antimicrobial activity against various important health microorganisms, and both compounds are documented to have a series of physicochemical problems that have limited their therapeutic use as specified below.

Curcumin is a hydrophobic polyphenol derived from *Curcuma longa* rhizomes that has a wide range of biological activities, including antimicrobial, anti-inflammatory, antioxidant, antitumor, antiviral, immunomodulatory, wound-healing, and anti-angiogenic activities, among others. However, its hydrophobicity, chemical instability, photosensitivity, low bioavailability, poor absorption in the gastrointestinal tract, and rapid decomposition have limited its therapeutic use [7,8,9,10,11,12,13,14,15,16]. On the other hand, essential oils correspond to a complex mixture of volatile secondary metabolites extracted from plants using different methods, commonly steam distillation. Essential oils are often composed of monoterpenoids, sesquiterpenoids, phenylpropanes, and low-molecular-weight aliphatic chains, among others [17,18,19]. Just like curcumin, they have a great variety of biological activities, which depend on the compounds that constitute each oil. The components mainly reported for *Lippia origanoides* essential oil are thymol and carvacrol [20,21,22,23,24,25,26,27,28], and various biological activities of this essential oil have been documented, such as antimicrobial, insecticidal, biocidal, larvicidal, antiparasitic, and antiviral activities [25,27,28]. However, like curcumin, essential oils also have a series of disadvantages that have limited their therapeutic use, including their high volatility and susceptibility to decomposition when directly exposed to heat, moisture, light, or oxygen; moreover, they can cause irritability or hypersensitivity reactions, such as on the skin, etc. [29].

Liposomes are considered promising drug carriers due to their capacity for controlled release of different molecules, cutaneous targeting, and transdermal administration, as well as their ability to preserve the activity and enhance the safety of molecules in antimicrobial therapy [30,31,32]. Liposomes are self-assembled vesicles composed of one or more phospholipid bilayers around an aqueous core that can encapsulate hydrophilic molecules in their aqueous interior and hydrophobic molecules in their lipid bilayers. They are biocompatible, biodegradable, non-toxic, non-immunogenic, and highly versatile [30,31,33,34,35]. The administration of bioactive compounds encapsulated in nanoparticles and liposomes is reported to be a viable strategy for resolving problems related to the use of antimicrobial agents that otherwise usually fail due to the acquisition of resistance by microorganisms [32]. Additionally, it has been indicated that the encapsulation of volatile natural compounds, such as essential oils, protects active compounds from environmental factors and decreases their volatility [36]. Therefore, the use of nanotechnology in conjunction with natural compounds could represent a promising future approach for the treatment of skin infections caused by microorganisms that have been reported to be resistant to various drugs. Taking the above into consideration, liposomes loaded with a mixture of curcumin and *Lippia origanoides* essential oil were prepared during the development of this research, and their antimicrobial and cytotoxic activities were evaluated in vitro.

## 2. Materials and Methods

### 2.1. Materials

The lipids used were 1,2-distearoyl-sn-glycero-3-phosphocholine (DSPC), purity > 99%, MW 790.145 g/mol and L-α-phosphatidylcholine from egg yolk (EYPC), purity > 99%, MW 768 g/mol (from Sigma Aldrich USA, Burlington, MA, USA). Curcumin (CUR) contains ≥94% curcuminoids, and ≥80% curcumin was obtained (from Sigma Aldrich USA, Burlington, MA, USA). The essential oil of *Lippia origanoides* (LEO), was acquired from the company Natuaroma (Armenia, Quindío, Colombia). Phosphate-buffered saline (PBS) was prepared as 137 mM NaCl, 2.7 mM KCl, 10 mM Na_2_HPO_4_, and 1.8 mM KH_2_PO_4_ at pH 7.4 [37]. All reagents were stored at −30 °C according to the manufacturer’s instructions. The transition temperatures for the lipids were DSPC: 55 °C and EYPC: −15 °C.

### 2.2. Chemical Characterization of the Essential Oil by Gas Chromatography–Mass Spectrometry (GC–MS)

Analysis of the chemical composition of *L. origanoides* essential oil was carried out in the Instrumental Analysis Laboratory of the Chemistry Program at the University of Quindío. Compounds in the oil were identified via gas chromatography–mass spectrometry (GC–MS) using Shimadzu equipment (injector: AOC-20i; sampler: AOC-20s; GC: GC-2010 Plus and GCMS-QP2010 Ultra (Sigma Aldrich USA, Burlington, MA, USA)). The NIST11 mass spectral library was used as a reference.

The 1 mg of sample was dissolved in 1 mL of chloroform (Supra Solv Merck 1-024321000 Sigma Aldrich USA, Burlington, MA, USA) to a concentration of 1 mg/mL. Then, 1 μL sample was injected in split mode (1:10 at 250 °C) with a flow of 1 mL/min into a Rxi^®^-5 ms column (RESTEK) (Cross-bond^®^ 5% diphenyl, 95% dimethyl polysiloxane (Sigma Aldrich USA, Burlington, MA, USA)) with dimensions of 30 m × 0.25 mm ID, 0.25 µm df. Chromatographic-grade helium (99.9999% purity) was used as the carrier gas along with the following temperature gradient: 1 min at 50 °C, increased at 3 °C/min to 110 °C, held for 1 min; increased at 2 °C/min to 200 °C, held for 1 min, and finally increased at 10 °C/min to 250 °C and held for 5 min. At 3 min after injection, the electron impact detector began scanning masses between 35 and 500 *m*/*z* every 0.30 s.

### 2.3. Preparation of Liposomes Loaded with Curcumin and L. origanoides Essential Oil

The liposomes were prepared using the thin-layer method [38]. Briefly, appropriate amounts of lipids and the compounds to be encapsulated, detailed in Table 2, were weighed and dissolved in chloroform, which was completely removed using rotary evaporation (Heidolph, Alpersdorfer, Schwabach, Germany) at 40 °C until a layer of dry lipids was obtained. The layer of dry lipids was stored at 4 °C to allow for complete removal of the solvent. Subsequently, the layer of dry lipids was hydrated with type I water using mechanical agitation through vortexing or sonication in a bath at 40 kHz (Branson, Ultrasonics™, CPX-952-238R, Sigma Aldrich USA, Burlington, MA, USA) for 1 h, and the resulting multilamellar liposome solution was extruded 35 times through a 100 nm polycarbonate filter using a mini-extruder (Avanti Polar Lipids, Alabaster, AL, USA) according to the manufacturer’s instructions. Finally, the liposomes were sterilized by filtration through a 0.45 µm PVDF membrane. For liposomes prepared with DSPC, hydration and extrusion were both carried out at 60 °C for 1 h.

### 2.4. Characterization of the Liposomes

The encapsulation efficiency of the curcumin and essential oil was evaluated using UV–VIS. In addition, the particle size, monodispersity, and zeta potential were evaluated through dynamic light dispersion, electrophoresis, and laser Doppler velocimetry.

The formula used to calculate encapsulation efficiency is the following:

$$Encapsulation\;efficiency=\frac{Amount\;of\;compound\;encapsulated}{Total\;amount\;of\;compound}*100$$

#### 2.4.1. Assessment of the Curcumin Encapsulation Efficiency

A curcumin calibration curve within a range of 0 to 20 µg/mL was prepared using a final concentration of 10% dimethyl sulfoxide (DMSO) and 0.5% Triton X-100, and each of the standards was read at 430 nm in triplicate on an EPOCH spectrophotometer (BioTek Instruments Inc., Winooski, VT, USA). The concentration expressed in µg/mL was solved based on the linear equation (y = mx + b) generated through Gen5 software v2.03.1, and an R^2^ of 0.99 was obtained.

#### 2.4.2. Assessment of the Encapsulation Efficiency of L. origanoides Essential Oil

The EYPC 20:2 liposomes were analyzed using GC–MS and the same conditions described in Section 2.2. The liposomes were dissolved in 90% HPLC-grade methanol (>99.9%). Liposomes were analyzed at a 1:10 dilution, and 2 μL of sample was injected in split mode (1:10 at 250 °C) with a flow of 1 mL/min; the column used was a Zebron™ ZB-5 ms (Phenomenex, Global Headquarters, Santa Clara, CA, USA).

Based on the GC–MS results, the protocol reported by Fadhil in 2014 was used, with some modifications, to detect thymol using UV–VIS [39]. This method is based on the reaction of thymol with 2,4-dinitrophenylhydrazine (2,4-DNPH) in the presence of potassium periodate (KIO_4_) in a basic medium, resulting in the formation of a violet-colored compound that absorbs at a wavelength of 570 nm. Briefly, solutions of 1 mM 2,4-DNPH, 5 mM KIO_4_, 0.5 M NaOH, 1 mg/mL curcumin in pure DMSO, and 100 µg/mL thymol in 90% methanol were prepared. To evaluate encapsulation efficiency, liposomes (EYPC 20:2) were used at a 1:10 dilution, and 25 µg/mL curcumin—approximately equivalent to the amount of curcumin inside the liposomes—was added to the mixture, represented by each point on the curve to account for its effects on the reading, which would be constant for all points on the curve. The compounds were added in the following order, according to Table 2: thymol, curcumin, KIO_4_, 2,4-DNPH, NaOH, and water. The tubes were mixed through vortexing and incubated at room temperature (approximately 22 °C) for 60 min; then, 100 µL was taken from the mixture for each point, in triplicate, and read using an EPOCH spectrophotometer (Biotek Instruments Inc. Winooski, VT, USA) at a wavelength of 560 nm (since curcumin addition shifted the maximum peak absorbance from 570 to 560 nm). The concentration expressed in µg/mL was solved based on the linear equation (y = mx + b) generated by Gen5 software v2.03.1, and an R^2^ of 0.99 was obtained.

#### 2.4.3. Assessment of the Particle Size and Zeta Potential of Empty and Loaded Liposomes

This characterization was conducted by Minerals Institute CIMEX at the Universidad Nacional de Colombia (Medellín, Colombia) as a contracted technical service. The samples were dissolved in type I water to a final lipid concentration of 0.05 mg/mL. The measurement was performed in a polystyrene cell using the ZetaSizer Nano ZS90 (Malvern Panalytical™ Grovewood Road Malvern, UK). This equipment measures particle sizes ranging from 0.3 nm to 5 µm and has the ability to determine the zeta potential of particles in the size range from 3.8 nm to 100 µm. This equipment utilizes dynamic light scattering (DLS) to determine the particle size from Brownian movement, while the zeta potential is measured using electrophoresis and laser Doppler velocimetry.

### 2.5. Evaluation of the Antimicrobial Activity In Vitro of Free and Encapsulated Compounds in EYPC 20:2 Liposomes against S. aureus ATCC 25923 

The in vitro antimicrobial activity was carried out using the plate microdilution technique and resazurin as a metabolic indicator [40]. To determine the maximum amount of solvent (DMSO) needed to dissolve curcumin and the essential oil, antimicrobial activity was first evaluated at concentrations ranging from 4 to 10%, and it was then used at a concentration of 5%, which does not affect growth against *S. aureus*.

Initially, the bacteria were seeded on Müeller Hinton agar (MHA), and an isolated colony was then taken and grown in Müeller Hinton broth (MHB) until reaching a concentration of 2–5 × 10^8^ colony forming units (CFUs)/mL corresponding to No. 0.5 on the McFarland scale (Optical Density at 600 nm (OD_600_): 0.078 to 0.1 in a 96-well plate, 100 µL); then, a 1:1000 dilution was made in MHB (working solution). Subsequently, a 10 µL solution of the compound to be evaluated (CUR or LEO) was added to a sterile 96-well plate, followed by the addition of 90 µL of the working solution of the microorganism. The plate sealed with plastic wrap was then incubated at 37 °C for 16 to 20 h. Curcumin was used at concentrations ranging from 2.5 to 300 µg/mL and LEO at concentrations ranging from 4.52 to 9040 µg/mL. All assays were performed in triplicate, and the following were used as controls: sterile medium and growth control of the microorganism. Resazurin (Acros Organics 418900050, Geel, Belgium Sigma Aldrich USA, Burlington, MA, USA) was used at a final concentration of 44 µM. The plates were incubated at 37 °C for 1 h, and a fluorescence reading was then performed using the Synergy HTX multimodal plate reader (BioTek Instruments Inc, USA) with excitation/emission at 579/584 nm (excitation filters: 565 ± 10 nm; emission: 600 ± 40). To analyze the results, the mean fluorescence of the blank (culture medium without bacteria) was subtracted from that of each sample, and the resulting fluorescence was used to calculate the growth percentage with respect to a growth control (% growth = bleached fluorescence/control bacteria * 100). This analysis was performed automatically using Gen5 v3.0 software. The minimum inhibitory concentration (MIC) is defined as the lowest concentration of the compound that inhibits 50% of bacterial growth [40]. Assays were performed in triplicate in three independent assays.

Assessment of liposome antimicrobial activity was conducted as described in the previous paragraph with the following modification: The bacterial working solution was prepared by adjusting the solution density according to No. 0.5 on the McFarland scale and diluting 2:1000 in MHB in concentration 2X. Next, 50 µL of the bacterial solution was added to the plate, followed by 50 µL of empty or loaded liposomes and their respective growth controls (50 µL bacterial solution + 50 µL type I water) as well as the medium control (50 µL MHB 2X + 50 µL type I water).

### 2.6. Assessment of Cytotoxic Activity in vitro

The cytotoxic activity of the essential oil and curcumin was evaluated using the VERO ATCC CCL 81 cell line, which is derived from the kidney of the african green monkey *Cercopithecus aethiops*. The methodology proposed by Toro et al. [40] was used with some modifications. The cells were cultured in Dulbecco’s Modified Eagle’s Medium (DMEM) (D5648 Sigma) supplemented with 10% bovine fetal serum (BFS) (CVFSVF00-01 Eurobio), 2 mM L-glutamine, 1X antifungal antibiotic 1X (10,000 units/mL of penicillin, 10,000 µg/mL of streptomycin, and 25 µg/mL of amphotericin B) (Sigma A5955) and incubated at 37 °C in a 5% CO_2_ atmosphere. A suspension of 10,000 cells was added to each well of sterile 96-well polystyrene microplates (NEST Ref. 701001) and incubated under the same conditions for 24 h to establish a cell monolayer of approximately 70–80% confluence. Subsequently, the plate medium was removed, and 100 µL of essential oil at concentrations ranging from 125 to 3000 µg/mL or curcumin at concentrations ranging from 10 to 50 µM (3.68 to 18.42 µg/mL) or 10 µL of EYPC 20:1 liposomes (corresponding to 15.7 µg/mL curcumin), all dissolved in the supplemented culture medium using a maximum DMSO concentration of 1% (except for the liposomes), was added to the wells. The treated cells were incubated at 37 °C in a 5% CO_2_ atmosphere for 24 h. All assays were performed in triplicate, and the following controls were used: medium control, vehicle control (1% DMSO or type I water), and cell growth control. After 24 h of incubation, the medium was removed from the wells, and 100 µL of resazurin dissolved in culture medium was added to a final concentration of 44 µM to each well of the plate, which was then incubated at 37 °C in a 5% CO_2_ environment for 4 h. Subsequently, fluorescence reading was performed using the Synergy HTX multimodal plate reader (BioTek Instruments Inc. Winooski, VT, USA) with excitation/emission at 579/584 nm (excitation filters: 565 ± 10 nm; emission: 600 ± 40).

To analyze the results, the mean fluorescence of the blank (control of cell culture media) was subtracted from that of each sample, and the cell viability percentage was calculated with respect to the control without compounds using the following formula: percentage of cell growth = (Fluorescence of the sample/Fluorescence of control cells) * 100. This analysis was carried out automatically using Gen5 software V. 3.0 (BioTek, Winooski, Vermont, USA) [40]. The cytotoxic concentration 50 (CC50) corresponds to the concentration that results in 50% reduction in growth or cell viability.

#### 2.6.1. Selectivity Index

The selectivity index (*SI*) of *L. origanoides* essential oil was calculated using the formula proposed by Cáceres et al, where positive values indicate higher selectivity against microorganisms than toxicity to VERO cells and negative values indicate higher toxicity to VERO cells than to bacteria [28].

$$SI=log\frac{\left[CC50\right]}{\left[MIC\right]}$$

#### 2.6.2. Statistical Analysis

Each experiment was conducted in triplicate through two or three independent assays and the means and standard deviations of the obtained data were analyzed. The D’Agostino–Pearson test or Shapiro–Wilk normality test was applied using GraphPad Prism software version 6.01 to assess whether the data followed a Gaussian distribution. Thereafter, analysis of variance (ANOVA) and Dunnett’s multiple comparison tests were applied for normal data and a non-parametric test (Kruskal–Wallis) for data that were not normally distributed, followed by Dunn’s multiple comparison test. A *p*-value of <0.05 was considered statistically significant.

The same software was used to calculate CC50 by transforming the data to a logarithmic scale and applying four-parameter logistic regression with variable slope using a log-type inhibitor vs. response model.

## 3. Results

### 3.1. Lippia Origanoides Essential Oil

The essential oil had slightly yellowish transparent coloration and a strong odor of citronella. The density was determined as 0.904 g/mL.

### 3.2. Chemical Characterization of the Essential Oil Using Gas Chromatography Coupled to Mass Spectrometry (GC–MS)

**Figure 1:**
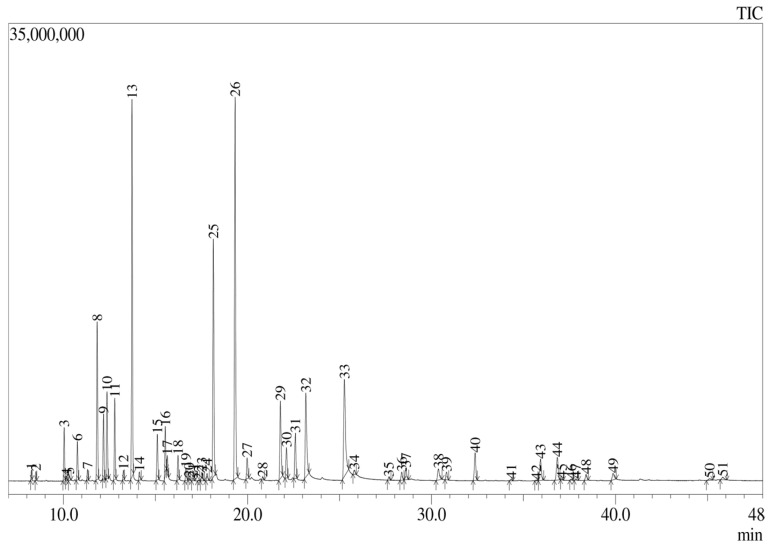
GC–MS chromatogram of the *L. origanoides* essential oil.

Figure 1 shows that a total of 51 peaks were observed, corresponding to compounds in the oil, from which the major components were found to be terpinen-4-ol (17.84%), γ-terpinene (14.72%), citronellal (10.08%), thymol (7.28%), and geraniol (5.28%), representing approximately 55% of the oil in total. The remainder were trace compounds or only present at <5%. The obtained chromatogram and list of compounds found are detailed in Table 1, which reports the peaks identified in the chromatogram of *L. origanoides* essential oil.

### 3.3. Preparation of Liposomes Loaded with Curcumin and L. origanoides Essential Oil

**Figure 2:**
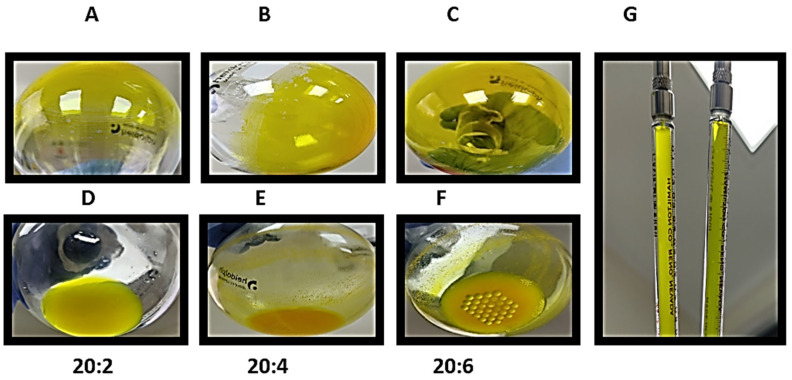
Photographs showing the preparation of different formulations of 10 mg/mL EYPC liposomes, empty and loaded with curcumin and *L. origanoides* essential oil in 20:2, 20:4, and 20:6 formulations. (**A**–**C**) thin layer of the 20:2, 20:4, and 20:6 formulations, respectively. Hydration process of the 20:2, 20:4, and 20:6 formulations is observed in (**D**–**F**) for liposomes before and after the extrusion process, where post-extrusion liposomes are more translucid (**G**).

For all the formulations, it was possible to observe the formation of thin layers, which were uniformly distributed on the balloon (Figure 2A–C). Following the addition of type I water, good hydration of the layer of dry lipids was observed in the 20:2 formulation without any precipitate (Figure 2D); nevertheless, for the 20:4 and 20:6 formulations, a large amount of precipitate material was observed (Figure 2E,F), which was removed before. Liposomes post-extrusion are more translucid (G).

**Figure 3:**
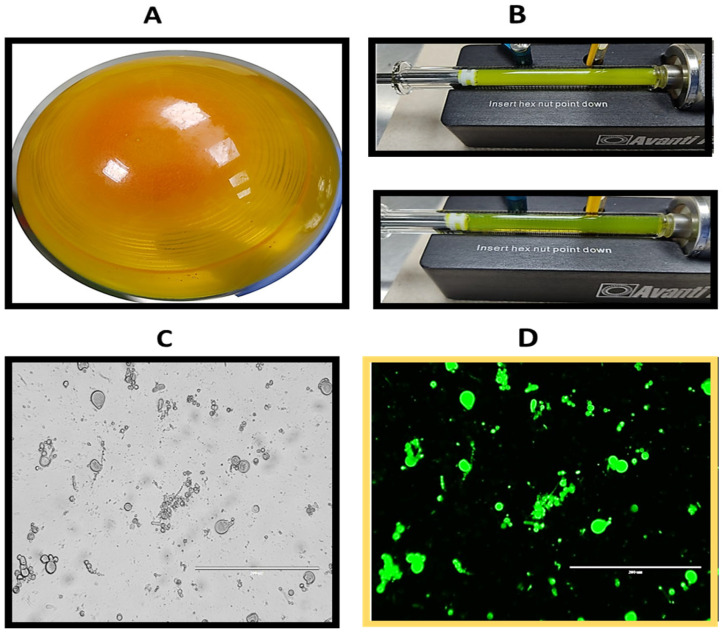
Photograph showing the preparation of DSPC liposomes with 20:2 formulation. (**A**) Formation of the thin layer of lipids and compounds. (**B**) Extrusion process. The liposomes in the syringe are observed at the beginning of the extrusion process (upper photograph) and after 35 passages (lower photograph). Photograph of loaded DSPC liposomes (**C**) before extrusion and (**D**) before extrusion viewed with the GFP filter.

Moreover, DSPC liposomes were prepared at a concentration of 10 mg/mL using the 20:2 formulation. After rotary evaporation, a uniform dry lipid layer was obtained (Figure 3A), which demonstrated greater resistance to detachment from the balloon during the hydration process in comparison to the lipid layer formed by EYPC. A milky yellow solution was obtained, with precipitated material present during the hydration process. Despite some difficulty caused by the amount of precipitate material that clogged the membrane and its respective filters, 35 passes were made through the polycarbonate membrane, with the temperature maintained at 60 °C throughout the process. After extrusion, the solution appeared to become more translucent (Figure 3B). An aliquot of the liposomes was taken before extrusion and observed under a microscope, revealing structures of various sizes and shapes (Figure 3C). Additionally, when viewed under the GFP filter, the apparent fluorescence of curcumin could be observed in these structures (Figure 3D).

### 3.4. Liposome Characterization

#### 3.4.1. Assessment of Curcumin Encapsulation Efficiency

A calibration curve with an R^2^ of 0.99 was obtained, and the equation of the curve was used to evaluate the curcumin encapsulation efficiency of each of the formulations prepared in Table 2. The values of encapsulated curcumin and the respective encapsulation efficiencies are shown in Table 2.

It was observed that the EYPC 20:2 formulation encapsulated approximately 100 µg/mL more curcumin than the 20:1 formulation, indicating that an excess of curcumin may be required for greater encapsulation inside bilayers. However, increasing the amount of curcumin in the 20:4 and 20:6 formulations did not result in more than 257.08 µg/mL being encapsulated, suggesting that the phospholipid bilayer became saturated. Furthermore, DSPC 20:2 liposomes encapsulated less curcumin than the EYPC 20:2 liposomes, with an approximate difference of 53 µg/mL. Based on these results, the EYPC 20:2 formulation was chosen for the remaining experiments.

#### 3.4.2. Assessment of the Encapsulation Efficiency of the *L. origanoides* Essential Oil

**Figure 4:**
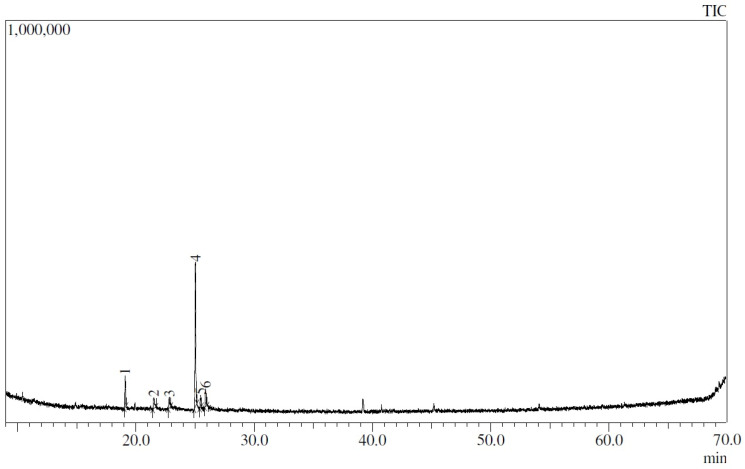
GC–MS chromatogram of EYPC 20:2 liposomes loaded with curcumin and *L. origanoides* essential oil.

It was noted that the highest peak found in the chromatogram of the EYPC 20:2 liposomes corresponds to thymol, with 63.34% (Figure 4). It is also observed that a peak corresponding to terpinen-4-ol (12.28%) was present; this is a major component of the oil but present in a lower proportion than thymol. No peak was observed corresponding to γ-terpinene, despite it being the second-most abundant compound in the essential oil. Table 3 shows all compounds of *L. origanoides* essential oil and percentage areas obtained in EYPC 20:2 liposomes.

Based on the results obtained from the previous chromatogram, thymol was used as a reference to evaluate the encapsulation efficiency of the essential oil. Initially, the conditions described by Fadhil [39] were evaluated, but it became necessary to modify the amounts of reagents used, increase the incubation time, and incorporate curcumin at a concentration similar to that found in the liposomes. After the protocol conditions were modified, a calibration curve was obtained in the range of 0 to 20 µg/mL with an R^2^ of 0.99. Using the equation of the line, it was determined that the amount of thymol in the diluted liposomes was 5.153 µg/mL, corresponding to a total of 51.53 µg/mL of thymol encapsulated in the liposomes. It should be noted that, for the formulation, 600 µg/mL of essential oil was used, which contains only 7.28% of thymol or an equivalent of 43.68 µg/mL. However, due to the higher concentration of encapsulated thymol compared to the original oil, the exact encapsulation efficiency cannot be determined. However, these results, in combination with the GC–MS chromatogram of the liposomes (Figure 4), suggest that the majority of compounds in the essential oil were not encapsulated. Only six compounds were found in the liposomes (Table 3), of which at least three were not identified in the initial report for the essential oil (Table 1). 

#### 3.4.3. Assessment of the Particle Size and Zeta Potential of Empty and Loaded Liposomes

The results of liposome characterization are summarized in Table 4. It was found that the empty EYPC 20:2 liposomes were slightly larger than the loaded liposomes, and no significant size variation was observed for either during the evaluation period. The zeta potential of empty liposomes was more negative (−27.3 mV) than that of loaded liposomes (−24.1 mV), but this value tended to become more neutral, and no liposome precipitation was observed. Additionally, the values obtained for the polydispersity index indicate that both types of liposomes are composed of highly homogeneous populations, indicating the success of the extrusion process and the application of an adequate number of passes through the 100 nm polycarbonate membrane.

Furthermore, it was observed that empty and loaded DSPC liposomes exhibit a high degree of heterogeneity in their particle sizes, as indicated by the elevated values for the polydispersity index. It was also noted that both types of liposomes exceeded 400 nm in size, despite being subjected to extrusion through a 100 nm polycarbonate filter. In addition, the loaded liposomes were significantly larger than the empty liposomes. Moreover, the zeta potential of the liposomes became less negative upon encapsulation of the compounds, which may have caused some particles to precipitate. Consequently, a second peak measuring 4569 nm was observed. Taking all these results into account, it is concluded that the EYPC liposomes are superior to the DSPC liposomes for encapsulating the assessed compounds.

### 3.5. Assessment of In Vitro Antimicrobial Activity

**Figure 5:**
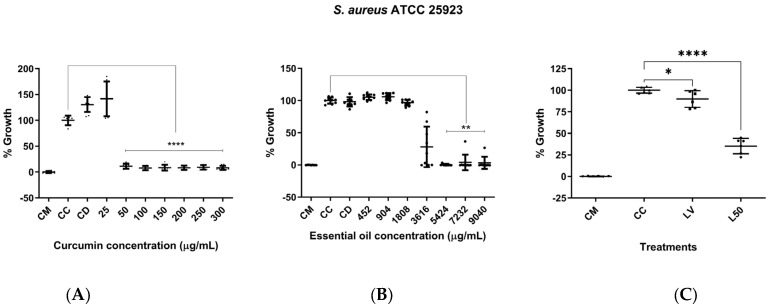
Assessment of antimicrobial activity of curcumin, L. origanoides essential oil, and EYPC 20:2 liposomes against S. aureus ATCC 25923. (**A**) Graph of the antimicrobial activity of curcumin at concentrations ranging from 25 to 300 µg/mL based on three independent assays (n = 3). (**B**) Graph of the antimicrobial activity of LEO at concentrations ranging from 452 to 9040 µg/mL based on three independent assays (n = 3). CM: medium control; CC: bacteria control; CD: solvent control (5% DMSO). (**C**) Graph of the antimicrobial activity of EYPC 20:2 liposomes. CM: medium control; CC: bacteria control; LV: empty liposome control (50 µL); L50: liposomes loaded with CUR and LEO (corresponding approximately to 128.54 µg/mL of CUR). Two independent assays were performed in triplicate (n = 2). *p* = * < 0.05; ** < 0.01; **** < 0.0001.

In terms of antimicrobial activity of curcumin against *S. aureus*, an MIC of 50 µg/mL was found. Statistically significant differences were observed compared to the bacteria control at concentrations of 50 µg/mL and above (*p* < 0.0001) (Figure 5A). Moreover, there was evidence of LEO antimicrobial activity against *S. aureus*, with an MIC of 4821.33 ± 1043.85 µg/mL. Likewise, statistically significant differences were found compared to the bacteria control at concentrations ranging from 5424 to 9040 µg/mL (*p* < 0.01) (Figure 5B). The EYPC 20:2 liposomes (50 µL, curcumin concentration of approximately 128.54 µg/mL), in turn, inhibited bacterial growth by approximately 65%, showing statistically significant differences compared to the bacteria control (*p* < 0.0001) (Figure 5C). On the contrary, DSPC 20:2 liposomes (50 µL, curcumin concentration of approximately 101.98 µg/mL) did not exhibit antimicrobial activity against *S. aureus.*

### 3.6. Assessment of In Vitro Cytotoxic Activity

**Figure 6:**
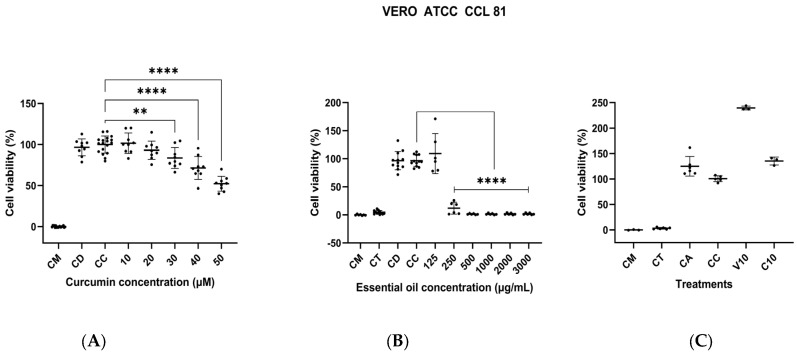
Assessment of the cytotoxic activity of curcumin, *L. origanoides* essential oil, and EYPC 20:1 liposomes against VERO cells ATCC CCL 81. (**A**) Plot of curcumin cytotoxic activity at concentrations ranging from 10 to 50 µM based on three independent assays (n = 3). CM: medium control; CC: cell control; CD: solvent control (1% DMSO). (**B**) Plot of LEO cytotoxic activity at concentrations ranging from 125 to 3000 µg/mL based on two independent assays (n = 2). CM: medium control; CC: cell control; CD: solvent control (1% DMSO); CT: Triton X-100 control (0.5%). (**C**) Plot of cytotoxic activity of EYPC 20:1 liposomes. V10: 10 μL of empty liposomes. C10: 10 μL of loaded liposomes. CM: medium control; CC: cell control; CA: liposome solvent control (type I water). CT: Triton X-100 control (0.5%). A single assay was performed (n = 1). *p* = ** < 0.01; **** < 0.0001.

A decrease in the viability of VERO cells was observed with 30 µM of curcumin, which was statistically significantly different compared to the cell control. However, none of the other curcumin concentrations decreased viability below 50% (Figure 6A), so the CC50 could not be calculated. Nevertheless, due to the decrease in cell viability and changes in morphology (rounded cells that detached from the plate), concentrations of curcumin higher than 30 µM (11.05 µg/mL); are not recommended. On the other hand, the cytotoxicity of the essential oil was evaluated, and statistically significant differences were found for the concentration of 250 µg/mL compared to the cell control (*p* < 0.0001) (Figure 6B). A CC50 of 186.8 µg/mL against this cell line was determined. Finally, the cytotoxic activity of EYPC 20:1 liposomes was evaluated in a single assay using a volume of 10 µL of liposomes per well, which is equivalent to a final curcumin concentration of 15.66 µg/mL (42.51 µM). No cytotoxicity against the VERO cells was observed for these liposomes. However, a substantial increase in metabolic activity was observed in cells treated with empty liposomes compared to the growth control (Figure 6C).

### 3.7. Selectivity Index

The selectivity index of the essential oil was determined, as shown in Table 5.

Based on the calculated value, it appears that our essential oil exhibits high toxicity against VERO cells.

## 4. Discussion

This research represents the first report on the co-encapsulation of curcumin and *L. origanoides* essential oil in EYPC and DSPC liposomes. Additionally, the antimicrobial and cytotoxic activities of these new vehicles were evaluated against *S. aureus* ATCC 25923 and VERO cells ATCC CCL 81.

The study initially focused on the chemical characterization of *L. origanoides* essential oil, with thymol and/or carvacrol previously reported as the main components in the literature [20,21,22,23,24,25,26,27,28,41,42,43,44,45,46,47,48,49,50,51,52,53,54,55,56]. Despite this, the essential oil used in this research was found to contain terpinen-4-ol (17.84%) and gamma-terpinene (14.72%) as the major components. It has been reported that differences in the chemical profiles of essential oils are due to variations in the extraction method and starting material, which are influenced by factors such as plant health, growth stage, habitat (including climate), soil factors, and harvest time [47,48].

The antimicrobial activity of the free compounds against *S. aureus* ATCC 25923 was evaluated using the broth microdilution technique and resazurin as a metabolic indicator. Curcumin and the *L. origanoides* essential oil were found to have antimicrobial activity at concentrations of 50 and 4821.33 µg/mL, respectively. Other researchers have also reported the antimicrobial activity of curcumin against different strains of *S. aureus* (both methicillin-sensitive and -resistant, MRSA) and other microorganisms using different methods such as broth microdilution [49], agar dilution [9], and broth microdilution [50], reporting MIC values different to those found in this research. This may be due to the differences in the technique selected to assess antimicrobial activity as well as in the concentrations or bacterial strains used [51]. Our results reaffirm the antimicrobial potential of curcumin against this potentially pathogenic species of Gram-positive bacteria and are in agreement with what has already been reported in the literature about the antimicrobial activity of the polyphenol curcumin [7,9,49,50]. Furthermore, it has been reported that the multiple mechanisms of action of curcumin may possibly help in promoting the resolution of infection and, for this same reason, reducing the likelihood of contributing to the selection of resistant bacteria [52].

Nonetheless, curcumin caused a decrease in cell viability when VERO cells were treated with concentrations as low as 30 µM (11.05 µg/mL), which is 4.5-fold lower than the MIC for *S. aureus*. This resulted in significant changes in cellular morphology, such as rounding of the cytoplasm and detachment of cells from the plate. However, other researchers have reported CC50 values for curcumin against VERO cells ranging from 30 µM to 1314.4 µM (484.2 µg/mL) [53,54]. This polyphenol has also been reported to have cytotoxic activity against other cell lines such as BHK-21, with a CC50 of 29.5 µM [55], and HT29 cells with a CC50 greater than 50 µM, causing detachment of cells from 40 µM upwards [56], with effects similar to those found in this study. The observed cytotoxic effects of curcumin may be explained on the basis of a previous report by Padilla et al. [55], who noted that curcumin can affect several cellular systems, including the cytoskeleton, ubiquitin–proteasome system, and nuclear and cellular morphology, possibly triggering apoptosis in BHK-21 and VERO cells [55]. While these results could cast doubt on the antimicrobial therapeutic potential of curcumin, due to its high in vitro toxicity, it is worth noting that curcumin has been reported as a safe molecule that is widely used in traditional medicine to treat different conditions and has not demonstrated toxicity in humans or animals, even at high doses [57].

It has been previously noted that the rise in antimicrobial resistance has led to more severe diseases being caused by pathogenic bacteria, prompting researchers to explore new alternative molecules against bacterial strains. Essential oils and their main chemical constituents are potential candidates as antimicrobial agents [48]. While our essential oil showed antimicrobial activity against *S. aureus*, it also exhibited high toxicity against VERO cells, as indicated by the calculated selectivity index. Previous studies have reported on the antimicrobial activity of LEO against *S. aureus* [23,26] and its cytotoxic activity against VERO cells, with CC50 values ranging from 31.4 ± 5.6 to 830 ± 20 µg/mL [21,28,41]. However, variations in these values may be expected, depending on the methodology used to assess antimicrobial and cytotoxic activities, as well as differences in the chemical composition of the oil and its major components [51,58].

Although liposomes and lipid vesicles loaded with curcumin have been produced by several research groups [59,60,61,62,63,64,65], co-encapsulation of curcumin together with *L. origanoides* essential oil has not been reported. For this study, two phospholipids (DSPC and EYPC) were chosen for producing liposomes, based on their phase transition temperature (T_M_). This parameter is important, as it can influence the encapsulation efficiency of the drug, stability during storage, and in vivo stability [38,66]. When the room temperature is higher than the T_M_, the phospholipids exhibit a fluid state, which is favorable for drug encapsulation during preparation due to their increased permeability to water [38,66]. However, in vivo, if the T_M_ is lower than body temperature, the liposome phospholipids become fluid, which can lead to premature drug release. Hence, phospholipids with a T_M_ higher than body temperature are frequently used to prepare stable liposomes for in vivo administration [38,66]. A high T_M_ also ensures good stability in terms of lipid bilayer rigidity, which is affected by fatty acid side chains, the degree of unsaturation, chain length, and polar headgroups [67]. Thus, DSPC was selected as one of the phospholipids to prepare the liposomes, as it contains two 18-carbon saturated fatty acids (18:0) and has a T_M_ of 55 °C. Nonetheless, EYPC was also used, with an approximate T_M_ of −15 °C, given that it has been reported to achieve fusogenicity (the ability of the liposomes to fuse with the external membrane of the bacteria [68] when the liposomes are in a fluid state [69]. Likewise, other factors such as the cholesterol content, pH, and the presence of different divalent cations also affect fusion between liposomes and bacteria [69]. In our research, T_M_ possibly played an important role in the antimicrobial activity assays, as activity was observed only in the fluid liposomes, i.e., those prepared with EYPC. However, our findings suggest that T_M_ had little influence on compound release in the in vitro diffusion assays, as statistically significant differences between the two types of liposomes in receptor medium B were observed only after 48 h.

The results show that the CUR-LEO/EYPC 20:2 formulation inhibits the growth of *S. aureus* ATCC 25923 by approximately 65%. In contrast, Ternullo et al. [65] reported that their curcumin-loaded liposomes exhibited antimicrobial activity against *S. aureus* ATCC BAA-1721 at a concentration of 100 µg/mL curcumin [44]. In our antimicrobial assays, we were able to reach a concentration 128.54 µg/mL curcumin in wells, a concentration even higher than that reported by Ternullo and colleagues [65]; however, we did not observe the same inhibition of *S. aureus* growth (almost 100%). Nevertheless, in their study, the empty liposomes, especially the cationic ones, also showed inhibitory activity against microorganism growth [44]. This raises doubts about whether the observed inhibitory effect was due to the encapsulated curcumin or the vehicle itself. Other studies have also shown that curcumin-loaded liposomes exhibit antimicrobial activity against *S. aureus* ATCC 29213, with MICs ranging from 3.5 to 14 µg/mL. However, only those liposomes containing a cationic lipid exhibited activity. The researchers also found that empty liposomes did not inhibit microorganism growth, and they concluded that their results support the idea of a synergistic effect between curcumin and cationic liposomes [70]. It has been reported that the charge and composition of liposomes modulate the fluidity and stability of their membranes, which in turn, has an impact on their interaction with bacteria [71]. Pathogenic bacteria have negatively charged cell walls, which makes them more likely to interact with cationic liposomes via electrostatic interactions [71]. Although neutral phospholipids were used in this investigation, the resulting liposomes had a slightly negative charge, which may have rendered the interactions with bacteria unfavorable and could partly explain the lack of antimicrobial activity of the DPSC liposomes. It has been reported that liposome–bacteria interactions are influenced not only by the characteristics of the liposomes themselves but also by other bacterial factors, such as membrane composition and surface patterns, which can affect the fusion and affinity with liposomes [69,71]. On the other hand, encapsulation of curcumin in EYPC liposomes appears to decrease its cytotoxicity. There was no reduction in cell viability even at a concentration of approximately 16 µg/mL (greater than 30 µM = 11.05 µg/mL). Furthermore, the observation of an increase in metabolic activity induced by empty liposomes against VERO cells is interesting. Further experiments are needed to generate hypotheses for this.

Regarding the encapsulation of compounds within liposomes, Kurien et al., reported that the solubility of curcumin in water is 0.6 µg/mL [72]. In the development of this research, it was possible to encapsulate 257.08 µg/mL curcumin within liposomes suspended in water, representing a 428-fold increase in solubility. As for the essential oil, only six compounds were detected in the chromatogram, of which at least three were not present in the initial report (Table 3). This suggests that some of the components may have volatilized or undergone chemical reaction during the process of liposome preparation, specifically during thin layer formation with the application of heat or use of solvents such as chloroform [47]. Therefore, thymol was used as a reference standard, as it was found in the highest proportion within the liposomes. However, its concentration was found to be higher than the initial concentration in the essential oil, supporting the previous hypothesis. Moreover, it has been shown that the biosynthetic pathway of thymol starts with γ-terpinene and involves a series of oxidation and dehydrogenation reactions catalyzed by specific enzymes [73]. This could explain why γ-terpinene was not found inside the liposomes but a higher amount of thymol was found than initially present in the essential oil, nevertheless, these are only hypotheses. Unfortunately, these findings made it impossible to determine the encapsulation efficiency of the essential oil, given the complete change in its composition. It is possible that liposomes are not the best vehicle to encapsulate essential oils, or at least not using the thin-layer method for production or any other processes involving heating. Despite this, several studies have reported the successful encapsulation of various essential oils in liposomes [30,74,75,76], but most do not report which compounds of the oil were encapsulated or whether their chemical composition changed after encapsulation.

In terms of the physicochemical characterization of the liposomes, the empty and loaded EYPC 20:2 liposomes were evaluated to determine their particle size, zeta potential, and polydispersity index (PI) during an 8-week period. It was found that loaded liposomes are smaller than empty liposomes. These results are similar to those reported by other researchers, who found that the curcumin incorporation reduces the size of liposomes, and curcumin is mainly located in the hydrophobic region of the EYPC liposomal bilayer, which has a significant impact on the vehicle stability [77]. In addition, it has been reported that the incorporation of essential oils into liposomes decreases their size, increases their fluidity, and results in reduced oxidation of the phospholipid bilayer [29]. On the other hand, zeta potential measurements have been reported to be predictors of the stability of colloidal systems. Higher absolute values of the zeta potential indicate greater stability of the particles, as there is less tendency for them to aggregate. Conversely, stability is considered low when the zeta potential value is around zero [29]. In this study, the zeta potential of the loaded liposomes was more negative (−24.1 mV) than that of the empty liposomes (−22.05 mV). However, for both types of liposomes, this value tended to become more neutral over time, which could affect their stability and lead to processes such as the flocculation, fusion, or coalescence of vesicles. Finally, considering the overall results of the particle size, polydispersity index, and zeta potential, it can be affirmed that liposomes remained stable during the 8-week period of evaluation, with a size range of 100 nm and a PI below 0.25. Despite the zeta potential becoming more neutral over time, no flocculation or coalescence processes occurred in these samples. Conversely, liposomes prepared with DSPC were significantly larger, had heterogeneous populations, and were much more unstable. Therefore, it can be concluded that EYPC liposomes are a better alternative for encapsulating CUR–LEO.

## 5. Conclusions

In this study, liposomes loaded with curcumin and *L. origanoides* essential oil were prepared, and their antimicrobial and cytotoxic activities were evaluated. Empty and loaded liposomes were successfully prepared using two different types of lipids (EYPC and DSPC. Notably, the CUR–LEO/EYPC formulation exhibited a nanometric size of 105.4 nm and a negative zeta potential of −24.1 mV, and was composed of a homogenous population of liposomes (PI = 0.114). Furthermore, this formulation was stable during the 8-week evaluation period and encapsulated more than 250 µg/mL curcumin. However, a complete alteration of the oil composition was detected after encapsulation in liposomes, rendering it impossible to calculate the encapsulation efficiency. The antimicrobial and cytotoxic activities of this formulation were evaluated, showing inhibition of *S. aureus* ATCC 25923 growth, while no cytotoxicity toward VERO cells was observed, but, rather, an increase in their metabolic activity. Our study demonstrates that nanotechnological tools can be used to significantly increase the solubility of natural compounds. Nevertheless, our findings suggest that liposomes may not be the optimal vehicle for encapsulating essential oils, as their composition can change after encapsulation. Alternative vehicles, such as nanoemulsions, may be more suitable for this purpose.

## Figures and Tables

**Table 1:** Report for peaks identified in the chromatogram of *L. origanoides* essential oil.

Compound	Retention Time (min)	Area %	Compound	Retention Time (min)	Area %
1. alpha-Phellandrene	8.252	0.28	27. L-alpha-Terpineol	19.977	1.03
2. alpha-Pinene	8.500	0.27	28. cis-Piperitol	20.816	0.13
3. Sabinene	10.030	1.78	29. Citronellol	21.786	3.88
4. beta-Pinene	10.159	0.14	30. 2-Isopropyl-5-methylanisole	22.118	1.46
5. 1-Octen-3-ol	10.304	0.24	31. 2-Isopropyl-5-methylanisole	22.597	2.27
6. beta-Myrcene	10.751	1.39	32. Geraniol	23.170	5.28
7. alpha-Phellandrene	11.305	0.37	33. Thymol	25.265	7.28
8. Terpinolene	11.825	5.83	34. Carvacrol	25.800	0.27
9. p-Cymene	12.171	2.41	35. gamma-Elemene	27.677	0.16
10. D-Limonene	12.362	3.44	36. alpha-Terpinyl acetate	28.382	0.46
11. Trans-beta-Ocimene	12.781	3.05	37. 2,6-Dimethyl 2,6-octadiene	28.606	0.71
12. beta-cis-Ocimene	13.257	0.32	38. Neryl acetate	30.375	0.89
13. gamma-Terpinene	13.722	14.72	39. (−)-beta-Elemene	30.819	0.45
14. p-Menth-8-en-1-ol, stereoisomer	14.120	0.37	40. Isocaryophyllene	32.370	1.7
15. Terpinolene	15.096	1.89	41. Humulene	34.335	0.09
16. beta-Terpineol	15.527	1.84	42. alpha-Amorphene	35.694	0.04
17. Linalool	15.635	0.53	43. alpha-Amorphene	35.927	1.41
18. 1-Octen-3-yl-acetate	16.218	0.99	44. gamma-Elemene	36.837	1.57
19. cis-4-(Isopropyl)-1-methylcyclohex-2-en-1-ol	16.651	0.57	45. alpha-Muurolene	37.081	0.06
20. 3-Tetradecanol acetate	16.813	0.07	46. beta-Bisabolene	37.596	0.06
21. 2,6-dimethyl -2,4,6-Octatriene	17.028	0.13	47. gamma-Muurolene	37.849	0.1
22. 3-Methyl-4-methylenebicyclo (3.2.1) oct-2-ene	17.337	0.02	48. (+)-delta-Cadinene	38.401	0.38
23. cis-4-(Isopropyl)-1-methylcyclohex-2-en-1-ol	17.518	0.41	49. Elemol	39.872	0.54
24. Isopulegol	17.799	0.36	50. alpha-Muurolol	45.120	0.18
25. Citronellal	18.142	10.08	51. Viridiflorol	45.829	0.26
26. Terpinen-4-ol	19.324	17.84	Total		100

**Table 2:** Curcumin encapsulation efficiency of different liposome formulations.

Formulation	Lipid (mg/mL)	Curcumin (µg/mL)	LEO (µg/mL)	Encapsulated Curcumin µg/mL ± SD	Encapsulation Efficiency (%) ± SD
Empty	10 (EYPC)	-	-	-	-
20:1		300	300	156.60 ± 2.71	52.2 ± 0.90
20:2		600	600	257.08 ± 0.99	42.85 ± 0.16
20:4		1200	1200	256.28 ± 1.43	21.36 ± 0.12
20:6		1800	1800	195.97 ± 8.96	10.89 ± 0.50
Empty	10 (DSPC)	-	-	-	-
20:2		580	580	203.97 ± 6.70	35.17 ± 1.15

Values correspond to the mean and standard deviation.

**Table 3:** Report for peaks from the chromatogram obtained from the EYPC 20:2 liposomes loaded with curcumin and *L. origanoides* essential oil.

Compound	Retention Time (min)	Area %
1. Terpinen-4-ol	19.099	12.28
2. Citronellol	21.509	6.89
3. Nitro-cyclopentane	22.816	5.23
4. Thymol	25.037	63.34
5. p-Cymen-7-ol	25.479	4.13
6. 4-Hydroxy-3-methylacetophenone	25.903	8.12

**Table 4:** Particle size, zeta potential, and polydispersity index of empty and loaded EYPC and DSPC liposomes containing curcumin and *L. origanoides* essential oil.

	Particle Size (nm) ± SD		Zeta Potential (mV) ± SD		Polydispersity Index (PdI)	
	Empty	Loaded	Empty	Loaded	Empty	Loaded
EYPC 0	122.3 ± 35.57	115.6 ± 38.32	−27.3 ± 4.89	−24.1 ± 11	0.213	0.114
1	125.7 ± 47.27	110 ± 34.38	−19.08 ± 13.5	−20.3 ± 10.3	0.161	0.141
2	136.8 ± 46.73	111.8 ± 33.18	−20.5 ± 8.95	−16.3 ± 7.29	0.089	0.148
8	113.7 ± 43.57	112.6 ± 31.65	−13.6 ± 5.45	−16.7 ± 5.38	0.124	0.057
DSPC (Upon sample reception)	Peak 1 (99.5%): 463.7 ± 286.2 Peak 2 (0.5%): 5292 ± 408.6	Peak 1 (80%): 690.4 ± 380.5 Peak 2 (20%): 4569 ± 904.6	−13.20 ± 4.4	−6.96 ± 3.57	0.230	0.577

**Table 5:** Selectivity index of the *L. origanoides* essential oil.

[MIC] (µg/mL)	[CC50] (µg/mL)	SI
4821.33	186.8	−1.41

## Data Availability

Data is contained within the article.
